# Evaluation of Two Beta-Tricalcium Phosphates with Different Particle Dimensions in Human Maxillary Sinus Floor Elevation: A Prospective, Randomized Clinical Trial

**DOI:** 10.3390/ma15051824

**Published:** 2022-02-28

**Authors:** Bruno Coelho Mendes, Rodrigo dos Santos Pereira, Carlos Fernando de Almeida Barros Mourão, Pietro Montemezzi, Anderson Maikon de Souza Santos, Jéssica Monique Lopes Moreno, Roberta Okamoto, Eduardo Hochuli-Vieira

**Affiliations:** 1 Department of Oral & Maxillofacial Surgery, Aracatuba School of Dentistry, Sao Paulo State University, Sao Paulo 16066-840, Brazil; andersonmaikon@hotmail.com (A.M.d.S.S.); hochuli@me.com (E.H.-V.); 2 Department of Oral & Maxillofacial Surgery, University of Grande Rio—UNIGRANRIO, Rio de Janeiro 25071-202, Brazil; dr.pereira@live.com; 3 Clinical Research Unit of the Antonio Pedro Hospital, Fluminense Federal University, Niteroi 24033-900, Brazil; m.montemezzi@libero.it; 4 Department of Dental Prosthesis, Aracatuba School of Dentistry, Sao Paulo State University, Sao Paulo 16066-840, Brazil; jessica_morenoo@hotmail.com; 5 Department of Human Anatomy, Aracatuba School of Dentistry, Sao Paulo State University, Sao Paulo 16066-840, Brazil; betaokamoto@gmail.com

**Keywords:** biomaterials, bone substitutes, sinus floor augmentation

## Abstract

This study aimed to compare two beta-tricalcium phosphates with different particle sizes in human maxillary sinuses lifting. The immunolabeling of cells for RUNX2 and VEGF were performed to evaluate the osteoblast precursor cells and the vascular formation after 6 months of bone repair. Ten maxillary sinuses were grafted with autogenous bone graft (Group 1), 10 were grafted with ChronOs^®^ (Group 2), and 10 were grafted with BETA-TCP^®^ (Group 3). After 6 months of bone healing, biopsies were obtained to assess the new bone formed by histomorphometric and immunohistochemical evaluation for RUNX2 and VEGF. The mean bone formation for Group 1 was 51.4 ± 17.4%. Group 2 presented 45.5 ± 9.9%, and Group 3 conferred 35.4 ± 8.0% of new bone formation. The RUNX2 offered low for Groups 1 and 2 with high cellular activity for osteoblast in Group 3. The VEGF immunolabeling was moderate for Groups 1 and 2 and intense for Group 3. In conclusion, it was possible to show that the bone substitutes evaluated in the present study presented suitable outcomes for bone regeneration, being an alternative for the autogenous bone graft in maxillary sinus bone height reconstruction.

## 1. Introduction

Alveolar bone resorption is a physiological, continual, and gradual process that occurs after tooth extraction and results in bone height or length deficiency [1,2,3,4]. In the posterior maxillary region, this issue represents a challenge for dental professionals in oral rehabilitation cases using dental implants [1,2]. Thus, with relevant results, maxillary sinus lifting surgery using biomaterials to reconstruct this area has been used to regenerate the height, allowing implant placement [3,4,5].

Autogenous bone graft is the “gold standard” bone substitute due to its unique possession of osteoinduction, osteoconduction, and osteogenic properties [6,7]. However, autogenous bone graft harvesting requires another surgical site and creates greater morbidity, even if harvesting is from the intraoral region [8,9]. As an alternative to autogenous bone grafts, other bone substitutes—such as allogenic grafts, xenografts, and alloplastic grafts—have been used with relevant results in the literature [10,11,12].

One of these biomaterials is the beta-tricalcium phosphate [β-TCP], a ceramic, alloplastic, biocompatible, and osteoconductive bone graft with rapid resorption that has been used in dental reconstruction during the years with similar results to autogenous bone graft in histometry and tridimensional volumetric changes [11,13,14,15,16,17,18,19,20,21,22,23]. However, histomorphometric studies showing this bioceramic as an alternative to human maxillary sinus bone height reconstruction are lacking in the literature.

Runt-related transcription factor 2 (RUNX2) is a protein responsible for regulating osteoblastic differentiation and bone formation. Its gene expression influences skeletal growth and bone turnover in the early stages of osteoblastic differentiation [19,20,21,22,23,24]. Vascular endothelial growth factor (VEGF) is one of the most important growth factors for the regulation of vascular development and angiogenesis. Since angiogenesis plays an important role in the osteogenesis process, VEGF influences skeletal development and postnatal bone repair. It is regulated by many factors, including growth and transcription factors, hormones, mechanical stimuli, and mainly hypoxia [23,25]. In order to understand the osteoblastic differentiation process during the bone repair period, the use of the specific protein marker runt-related transcriptor factor 2, in immunohistochemical analysis complements the histomorphometric findings, as well as the vascular endothelial growth factor, highlights the event of vascular neoformation [19,20].

Therefore, the present study aimed to compare two different brands of β-TCPs (ChronOs^®^ and BETA-TCP^®^) in human maxillary sinuses lifting using histomorphometric and immunohistochemical evaluation.

**Hypothesis** **0** **(H0).** *There is no difference in the new bone formation between ChronOS^®^ and BETA-TCP^®^.*

## 2. Materials and Methods

### 2.1. Human Subjects

**Figure 1:**
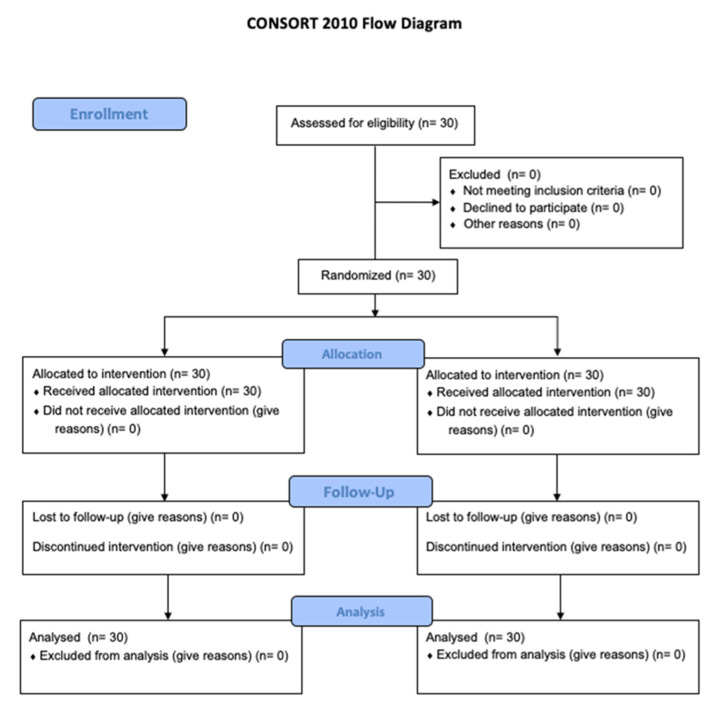
CONSORT diagram of the patient’s allocation by randomization.

This research was approved by the ethical committee of the Aracatuba School of Dentistry-UNESP through the Brazil Platform, protocol number 91334718.8.0000.5420. The present study was also registered in the international clinical trials registry platform with the number U1111-1251-7170. Quality assessment was carried out according to the CONSORT Statement’s RCT checklist (Figure 1) [26]. Patients were treated according to the principles incorporated in the Declaration of Helsinki.

### 2.2. Number of Samples to Be Evaluated

The necessary sample size for each group was determined by a statistical power test performed at the website “http://calculoamostral.bauru.usp.br/” (last accessed on 12 July 2020) using data from previous research [19]. The results showed a minimum of 8 maxillary sinuses for each group when the test was applied with a power of 80%, a mean difference of 15.1, and standard deviation of 9.9, and a significance level of 0.05 conducted in a single tail test.

### 2.3. Randomization

Each graft site was evaluated by a clinical assistant and randomization, performed at the website “http://www.random.org” (last accessed on 27 November 2020), determined the biomaterials used at the graft site for each patient. 

### 2.4. Inclusion and Exclusion Criteria

The inclusion criteria were as follows: atrophic posterior maxillary region and residual bone height less than 5 mm requiring bone augmentation rehabilitation with dental implants in healthy patients. The following were excluded: smokers, patients with the uncontrolled systematic disease, maxillary sinus diseases, those who received radiotherapy and chemotherapy, and patients with an inadequate amount of bone in the mandible symphysis where the autogenous bone graft was harvested. In order to analyze the maxilla, as well as the mandible symphysis, cone beam computed tomography was performed. The study’s primary outcome was the elevation of the sinus floor with bone substitutes, and the secondary outcome was the installation of implants for oral rehabilitation. 

### 2.5. Determination of the Groups Evaluated

After the inclusion and exclusion criteria, 30 patients were selected with unilateral maxillary sinus requiring to be grafted for posterior dental implant rehabilitation. The present study was performed after obtaining informed consent from all patients and the formation of the three study groups: 10 maxillary sinuses grafted with autogenous bone graft as Group 1 (control group), 10 maxillary sinuses grafted with ChronOS^®^ (DePuy Synthes^®^, Paoli, CA, USA) β-TCP as Group 2, and 10 maxillary sinuses grafted with BETA-TCP^®^ (Bionnovation^®^, Biomedical, Sao Paulo, Brazil) as Group 3.

β-TCP ChronOs^®^ (DePuy Synthes^®^, Paoli, CA, USA) is a biomaterial with homogenous porous structure, with particles from 0.5 mm to 0.7 mm in size [19,20,21,22].

The BETA-TCP^®^ (Bionnovation^®^, Biomedical, Sao Paulo, Brazil) is a β-TCP with microporosity ranging from 0.1 to 0.5 mm in diameter and composed of calcium hydroxide, phosphoric acid, and approximately 95% of calcium phosphate proportion in its formula with an absence of cytotoxicity [23]. In the present research, the authors referred to “Bion” as BETA-TCP^®^ to facilitate the interpretation of our results by the readers.

### 2.6. Surgical Procedures

The same surgeon performed all surgical procedures under local anesthesia using Mepivacaine 2% with adrenalin 1:100.000 (DFL^®^, Rio de Janeiro, Brasil). According to Pereira et al. [27], the autogenous bone graft blocks were harvested and milled with a bone crusher. The wound was sutured using a 5.0 polyglactin resorbable suture (Ethicon^®^, Johnson & Johnson, Sao Paulo, Brazil). According to Boyne & James [5], Maxillary sinus height reconstruction was performed and grafted with one of the three bone substitutes reported. 

Five hundred milligrams of paracetamol (Tylenol^®^, Johnson & Johnson, Sao Paulo, Brazil) 4 times per day for 2 days and 500 mg of amoxicillin (Eurofarma^®^, Sao Paulo, Brazil) 3 times per day for 1 week were prescribed in order to reduce pain and infection, respectively. 

### 2.7. Biopsy Sample Harvesting

After 6 months of bone healing, bone biopsies were harvested using a 3.0 mm × 15 mm trephine burr in the same direction as the dental implant placement. The samples were packaged in a 10% formalin solution (Sigma-Aldrich, St Louis, MO, USA) for 48 h, keeping the apical-coronal position, after that, they were subjected to washing for 24 h in running water. Then, the decalcification was carried out by submerging the samples at 10% ethylenediamine tetra-acetic acid (Sigma-Aldrich, St Louis, MO, USA) for 5 weeks, with weekly substitution during all laboratory processes as recommended by Pereira et al. [19]. Subsequently, after decalcification, the diaphanization process was carried out following the sequence of alcohols and xylol described: 70% alcohol—1 h; 80% alcohol—1 h; 90% alcohol—13 h; 95% alcohol—1 h; 100% alcohol (first stage)—1 h; 100% alcohol (second stage)—1 h, 100% alcohol (third stage)—1 h; alcohol associated with xylol—20 min; pure xylol (step I)—20 min; and xylol (step II)—20 min. Afterward, the samples were embedded in paraffin blocks. They were subjected to semi serial sections of 5 µm by a blade attached to a microtome for later staining with hematoxylin and eosin (Thermo Fisher Scientific, Waltham, MA, USA). 

### 2.8. Histomorphometric Analysis

One researcher observed t in three regions, new bone (2 mm above the samples the residual bone), intermediate, and apical regions (2 mm bellow of the Schneiderian membrane), using a light microscope in a 12.5 × magnification attached to a microcompute and captured using an image camera (LeicaR^®^ DC 300F microsystems Ltd., Heerbrugg, Switzerland). The images were recorded as a TIFF file and evaluated using ImageJ 150e software (National Institutes of Health, Bethesda, MD, USA). New bone formation, connective tissue, and remaining biomaterial were assessed according to Bonardi et al. [20], with the results expressed in percentages.

### 2.9. Immunohistochemistry Evaluation

For the immunohistochemical procedure, polyclonal primary antibodies from goats were used for RUNX2 (goat anti-RUNX2, Santa Cruz Biotechnology, SC8566, Santa Cruz, CA, USA) and VEGF (goat anti-VEGF, Santa Cruz Biotechnology, SC1881, Santa Cruz, CA, USA). Secondary antibodies were used against biotinylated goat IgG (anti-goat IgG-HRP, PIERCE, Jackson Immunoresearch Laboratories, West Grove, PA, USA). The detection method was performed by immunoperoxidase (Sigma-Aldrich, St Louis, MO, USA), and 3,3 diaminobenzidine (DAB, Sigma, St. Louis, MO, USA) was used as chromogen followed by nuclear counterstaining with Harris hematoxylin (Thermo Fisher Scientific, Waltham, MA, USA). Control procedures were performed by omitting primary antibodies (negative control). The blocking of endogenous peroxidase activity was performed with 30% oxygenated water (perhydrol 30% H2O2—MERCK, Rio de Janeiro, Brazil) for 45 min. The subsequent process of antigenic recovery by citrate buffer (Sigma-Aldrich, St Louis, MO, USA) (pH 6.0–55 °C) for later incubation during 18 h at 4 °C with specific primary antibodies for RUNX2 (goat anti-RUNX2—Santa Cruz Biotechnology, SC8566, Santa Cruz, CA, USA) which is responsible for the early stages of osteoblast differentiation [28] and VEGF (vascular endothelial growth factor; goat anti-VEGF—Santa Cruz Biotechnology, SC1881, Santa Cruz, CA, USA) which is responsible for promoting the migration of endothelial cells [29]. After 18 h, the slides were washed with PBS (Sigma-Aldrich, St Louis, MO, USA) (three washes) and incubated with a second biotinylated antibody (Thermo Fisher Scientific, Waltham, MA, USA) for an additional 1 h at room temperature. Moreover, washing was performed in PBS and incubated with the avidin–biotin complex (StreptABComplex/HRP-Vector, San Francisco, CA, USA) for 45 min. The immunohistochemical evaluation was performed by a single researcher with advanced training, according to Pereira et al. [30] and Bonardi et al. [20] using a semi-quantitative approach with the following scores: 0 for the absence of staining, 1 for low, 2 for moderate, and 3 for intense.

### 2.10. Statistical Analysis

The results of the histomorphometric evaluation underwent the Kolmogorov–Smirnov test to determine homoscedasticity. Due to the normal distribution, an ANOVA statistical test was performed, and a priori *p*-value < 0.05 was considered significant. (GraphPad Prism 8, San Diego, CA, USA)

## 3. Results

Thirty patients (18 females and 12 males) ages, ranging from 50 to 70 years old, underwent unilateral posterior maxillary bone height reconstruction using three different types of biomaterials.

### 3.1. Histomorphometric Outcomes

**Figure 2:**
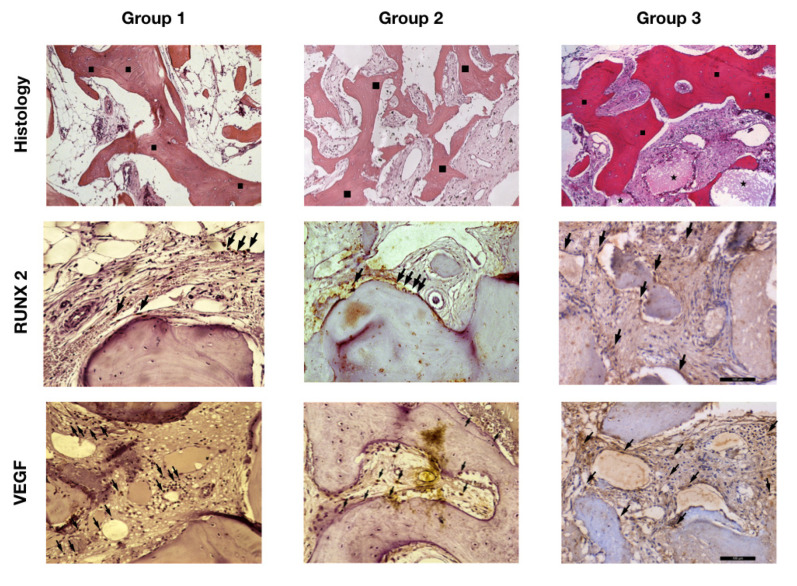
Image showing the histological section for histology evaluation and immunolabeling for RUNX2 and VEGF for the groups evaluated. (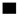) Lamellar bone formation. (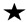) Bion particles remaining. (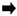) Immunolabeling cells for RUNX2 and VEGF. [Magnification, ×12.5].

**Figure 3:**
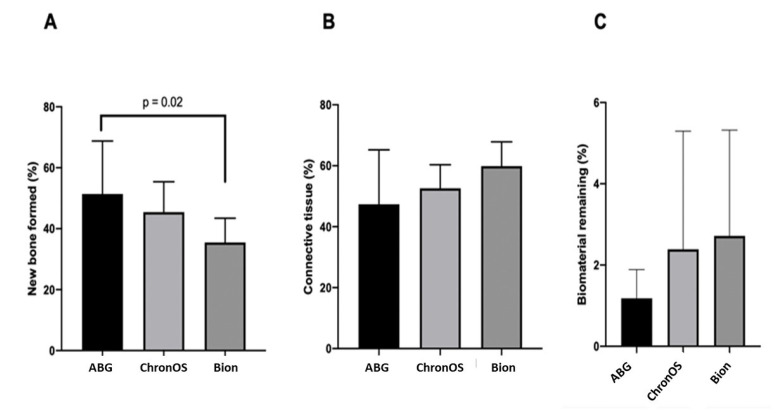
Graphic showing the histomorphometric outcomes for new bone formation (**A**), connective tissue (**B**), and biomaterial remaining (**C**) after 6 months in the human maxillary sinus augmented with the three bone substitutes studied.

Group 1 presented samples showing regions with lamellar bone formation in an organized matrix with the presence of osteocytes as well as the presence of osteoblasts in its periphery. The connective tissue presented as well cellularized with vascular formation. The mean bone formation for Group 1 was 51.4 ± 17.4%, the mean connective tissue was 47.4 ± 17.9%, and the particles of biomaterial remained was the smallest among the groups evaluated, with 1.2 ± 0.70%. Group 2 presented considerable mature newly-formed bone tissue in well-cellularized and vascularized connective tissue. The new bone formation rate was 45.5 ± 9.9%; the connective tissue and biomaterial remaining mean was 52.1 ± 7.7% and 2.4 ± 2.9%, respectively. Group 3 showed a lamellar bone formation as well as the presence of a connective tissue widely vascularized and cellularized. The histometry for the present group was 36.6 ± 8.0% of the rate of new bone formation; the median for connective tissue was 60.5 ± 7.9%, and the biomaterial remaining was 2.9 ± 2.6% (Table 1; Figure 2 and Figure 3). According to the ANOVA test, the comparison among the three groups evaluated showed a statistical difference (*p* = 0.02) for the new bone formed. The post hoc test identified difference between Groups 1 and 3 (*p* = 0.02) and no difference between Groups 1 and 2 (*p* = 0.53) as well as Groups 2 and 3 (*p* = 0.19). The connective tissue (*p* = 0.08) and the biomaterial remaining (*p* = 0.30) for the three groups demonstrated with no statistical difference. Thus, the null hypothesis was accepted.

### 3.2. Immunohistochemistry Outcomes

The immunostaining for RUNX2 was low “1” for Groups 1 and 2, with few osteoblasts stained at the periphery of the newly formed bone and lamellar bone; however, there was moderate “2” immunostaining for RUNX2 in Group 3, with the presence of pre-osteoblasts and osteoblasts stained in the connective tissue and periphery of the new bone formed. VEGF was moderate “2” for Groups 1 and 2 with the immunostaining for endothelial cells as well as osteoblasts in the mature bone matrix and in the connective tissue at the periphery of vascular neoformations. However, Group 3 presented intense “3” immunostaining in vascular regions adjacent to the biomaterial (Table 2; Figure 2). Thus, the immunohistochemical outcomes corroborate with histological results, demonstrating that Groups 1 and 2 were in the remodeling phase of bone healing. Additionally, it was possible to observe in Group 3 the presence of cell activity for the two proteins evaluated, indicating that it would require more time for the new bone regeneration.

## 4. Discussion

β-TCP is an osteoconductive biomaterial where the particle’s size directly influences bone healing and neovascularization [11,17,19,20]. According to Karageorgiou and Kaplan [31], the minimum size of the particle’s micropores particle is approximately 100 µm. This particularity allows for the approach of vascular and osteoblastic cells. Moreover, their study reported that a micropore size of roughly 300 µm would be ideal for optimizing bone healing. In contrast, a smaller size would predispose the tissue to hypoxia, leading to cartilage formation instead of bone [31].

Other researchers have also reported that microporosity contributes to an increase in surface area, as well as the adsorption of osteoinductive proteins [31,32,33]. Two biomaterials with different particle sizes and diameters were compared in the present study. ChronOS has particles sizes ranging from 500 to 700 µm and micropores with 10 µm of diameter [21,22], while the Bion of Group 3 has particle sizes of 100 to 500 µm and micropores ranging from 0.07 to 0.11 µm in diameter [23]. The differences of porosity presented by both biomaterials evaluated in the present study were verified in the new bone formed after 6 months of bone repair. Thus, the null hypothesis purpose was denied.

The new bone formation observed in Group 1 can be attributed to the presence of growth factors, as well as osteogenic elements present in the autogenous bone graft [33]. As a result, an autogenous bone graft can be considered the most reliable biomaterial for maxillary sinus bone height reconstruction. This result of the present study kept in line with the current literature [34,35,36].

The β-TCPs evaluated showed a different amount of bone formation being statistically significant when β-TCP of Group 3 was compared with the control group instead of ChronOS which presented relevant results in posterior maxillary bone height reconstruction. However, autogenous bone graft continues to be biologically superior. Group 3 of the present study showed more biomaterial particles remaining than Group 2 after 6 months of bone healing. Besides this, the Bion demonstrated less lamellar bone formation than autogenous bone graft, which suggests that bone formation is different in speed than biomaterial resorption.

Bonardi et al. [20] evaluated ChronOS compared with BioOss in maxillary sinus bone reconstruction and found more bone formation with β-TCP. However, it is essential to point out that the resorption rates of BioOss are low, which can influence new bone formation. The combination of ChronOs with autogenous bone graft in 1:1 ratio showed similar bone formation as autogenous bone graft alone, which indicates that this combination is a viable alternative, especially in cases of limited bone quantity on the donor site when the use of autogenous bone is necessary. The present study results also demonstrated the low presence of biomaterial remaining for the three groups evaluated, demonstrating the osteoconductive potential of β-TCP. Besides this, the current study results were similar to those of Szabò et al. [11], which found 36.47% of the bone area using β-TCP; thus, both biomaterials studied have suitable outcomes in the new bone formation and are promising for dental implant placement.

Regarding the volumetric changes in β-TCP, Gorla et al. [17] demonstrated results similar to those of autogenous bone grafts, and the results indicated the possibility of up to 40% overcorrection of the biomaterial grafting for the formation of the minimum amount of bone required for dental implant placement. For biomaterials with particle sizes smaller than 0.1 mm, this addition can be challenging to perform due to the technique’s sensitivity to the filling and the high flow of the material when in contact with blood and its dispersion. Moreover, this overcorrection can lead to hypoxia, making a complex environment for vascularization and bone formation [31].

The low immunostaining for RUNX2 in Groups 1 and 2 demonstrates a decrease in the osteoblast differentiation process; however, it tends to be intense for Group 3, mainly in the regions next to the bone matrix. Therefore, it is possible to suggest that it is probable that Group 3 will increase the new bone formation or the lamellar bone type since osteoblast differentiation still occurs. VEGF immunostaining was balanced between Groups 1 and 2 with more activity in Group 3. This outcome demonstrates the presence of cellular activity for osteoblast differentiation and vascular formation after 6 months of bone repair [37]. Thus, the immunostaining obtained in the present study could confirm that the Bion group needs more time to heal the bone graft but with a suitable pathway, as demonstrated by the lamellar formation in the histology sections.

Synthetic calcium/phosphate biomaterials are used to fill bone defects because they present a satisfactory biological behavior and safety in relation to allogeneic and xenogeneic grafts, in addition to presenting a three-dimensional arrangement that favors the processes of angiogenesis and osteogenesis, acting as an osteoconductive material. Despite this, the particle size and porosity must be carefully evaluated, considering the size of the defect to be reconstructed [1,11,14,17,19,20].

As a limitation for the present study, there is no evaluation of the dental implants after the rehabilitation procedure in function. However, the histomorphometric and immunostaining patterns of the test groups showed promising results compared to the control group. Besides this, biopsies with more bone healing time can be performed to evaluate in the long term if the Bion can improve the histomorphometric results.

## 5. Conclusions

The present prospective clinical research evaluated two different β-TCPs for bone regeneration in maxillary sinus elevation. The histomorphometry patterns showed superior new bone formation in Group 1 (control/autogenous graft) when compared to Group 3. However, similar results were obtained between β-TCPs (Groups 2 and 3). In addition, the immunolabeling of the proteins evaluated in Group 3 indicates that it would require more time for the new bone regeneration than the other groups. To assess this result, the authors suggest more physicochemical and characterization studies comparing cells’ activities, such as in osteoblasts, to explore/identify the difference observed in the histomorphometry and immunostaining from the present study. Other clinical studies with long-term evaluations of the bone substitute from Group 3 can help explain the differences found between Group 1 and Group 3. In conclusion, the present study showed that the bone substitutes presented relevant results for bone regeneration as an alternative to the autogenous bone graft in maxillary sinus bone height reconstruction.

## Figures and Tables

**Table 1:** Histometric outcomes of the three groups after 6 months of bone repair.

Groups	New Bone Formed (% SD)	Connective Tissue (% SD)	Biomaterial Remaining (% SD)
1	51.4 ± 17.4 *	47.4 ± 17.9	1.2 ± 0.7
2	45.5 ± 9.9	52.1 ± 7.7	2.4 ± 2.9
3	36.6 ± 8.0 *	60.5 ± 7.9	2.9 ± 2.6

* Occurred statistically significant difference (*p* < 0.05).

**Table 2:** Immunohistochemical outcomes for RUNX2 and VEGF of the three groups after 6 months of bone repair.

	RUNX2	VEGF
Group 1	+	++
Group 2	+	++
Group 3	++	+++

+ for low, ++ for moderate, and +++ for intense.

## Data Availability

Not applicable.

## References

[1] Pereira R., Menezes J., Bonardi J., Griza G., Okamoto R., Hochuli-Vieira E. (2018). Comparative study of volumetric changes and trabecular microarchitecture in human maxillary sinus bone augmentation with bioactive glass and autogenous bone graft: A prospective and randomized assessment. Int. J. Oral Maxillofac. Surg..

[2] Del Fabbro M., Corbella S., Weinstein T., Ceresoli V., Taschieri S. (2012). Implant survival rates after osteotome-mediated maxillary sinus augmentation: A systematic review. Clin. Implant Dent. Relat. Res..

[3] Starch-Jensen T., Aludden H., Hallman M., Dahlin C., Christensen A.-E., Mordenfeld A. (2018). A systematic review and meta-analysis of long-term studies (five or more years) assessing maxillary sinus floor augmentation. Int. J. Oral Maxillofac. Surg..

[4] Tatum H. (1986). Maxillary and sinus implant reconstructions. Dent. Clin. N. Am..

[5] Boyne P.J., James R.A. (1980). Grafting of the maxillary sinus floor with autogenous marrow and bone. J. Oral Surg..

[6] Mish C. (2008). Maxillary sinus anatomy, pathology and graft surgery. Contemporary Implant Dentistry.

[7] Wood R.M., Moore D.L. (1988). Grafting of the maxillary sinus with intraorally harvested autogenous bone prior to implant placement. Int. J. Oral Maxillofac. Implant..

[8] Hench L.L. (1998). Biomaterials: A forecast for the future. Biomaterials.

[9] Sbordone L., Toti P., Fabris G.B.M., Piombino P., Guidetti F. (2009). Volume changes of autogenous bone grafts after alveolar ridge augmentation of atrophic maxillae and mandibles. Int. J. Oral Maxillofac. Surg..

[10] Moy P.K., Lundgren S., Holmes R.E. (1993). Maxillary sinus augmentation: Histomorphometric analysis of graft materials for maxillary sinus floor augmentation. J. Oral Maxillofac. Surg..

[11] Szabó G., Huys L., Coulthard P., Maiorana C., Garagiola U., Barabás J., Németh Z., Hrabák K., Suba Z. (2005). A prospective multicenter randomized clinical trial of autogenous bone versus beta-tricalcium phosphate graft alone for bilateral sinus elevation: Histologic and histomorphometric evaluation. Int. J. Oral Maxillofac. Implant..

[12] Yildirim M., Spiekermann H., Handt S., Edelhoff D. (2001). Maxillary sinus augmentation with the xenograft Bio-Oss and autogenous intraoral bone for qualitative improvement of the implant site: A histologic and histomorphometric clinical study in humans. Int. J. Oral Maxillofac. Implant..

[13] Artzi Z., Weinreb M., Givol N., Rohrer M.D., Nemcovsky C.E., Prasad H.S., Tal H. (2004). Biomaterial resorption rate and healing site morphology of inorganic bovine bone and beta-tricalcium phosphate in the canine: A 24-month longitudinal histologic study and morphometric analysis. Int. J. Oral Maxillofac. Implant..

[14] Bettach R., Guillaume B., Taschieri S., Del Fabbro M. (2014). Clinical Performance of a Highly Porous Beta-TCP as the Grafting Material for Maxillary Sinus Augmentation. Implant Dent..

[15] Bohner M. (2000). Calcium orthophosphates in medicine: From ceramics to calcium phosphate cement. Injury.

[16] Ghosh R., Sarkar R. (2016). Synthesis and characterization of sintered beta-tricalcium phosphate: A comparative study on the effect of preparation route. Mater. Sci. Eng. C Mater. Biol. Appl..

[17] Gorla L.D.O., Spin-Neto R., Boos F., Pereira R.D.S., Garcia-Junior I., Hochuli-Vieira E. (2015). Use of autogenous bone and beta-tricalcium phosphate in maxillary sinus lifting: A prospective, randomized, volumetric computed tomography study. Int. J. Oral Maxillofac. Surg..

[18] Lu J., Descamps M., Dejou J., Koubi G., Hardouin P., Lemaitre J., Proust J.-P. (2002). The biodegradation mechanism of calcium phosphate biomaterials in bone. J. Biomed. Mater. Res..

[19] Pereira R., Gorla L.F.D.O., Boos F., Okamoto R., Júnior I.G., Hochuli-Vieira E. (2017). Use of autogenous bone and beta-tricalcium phosphate in maxillary sinus lifting: Histomorphometric study and immunohistochemical assessment of RUNX2 and VEGF. Int. J. Oral Maxillofac. Surg..

[20] Bonardi J.P., Pereira R.D.S., Lima F.B.D.J.B., Faverani L.P., Griza G.L., Okamoto R., Hochuli-Vieira E. (2018). Prospective and Randomized Evaluation of ChronOS and Bio-Oss in Human Maxillary Sinuses: Histomorphometric and Immunohistochemical Assignment for Runx 2, Vascular Endothelial Growth Factor, and Osteocalcin. J. Oral Maxillofac. Surg..

[21] Pereira R.D.S., Boos F.B., Gorla L.F.D.O., Garcia I.R., Okamoto R., Hochuli-Vieira E. (2016). Maxillary Sinus Elevation Surgery with ChronOS and Autogenous Bone Graft: Analysis of Histometric and Volumetric Changes. Int. J. Periodontics Restor. Dent..

[22] Walsh W.R., Vizesi F., Michael D., Auld J., Langdown A., Oliver R., Yu Y., Irie H., Bruce W. (2008). β-TCP bone graft substitutes in a bilateral rabbit tibial defect model. Biomaterials.

[23] Milhan N.V.M., Carvalho I.C.S., do Prado R.F., de Sousa Trichês E., Camargo C.H.R., Camargo S.E.A. (2017). Analysis of indicators of osteogenesis, cytotoxicity and genotoxicity of an experimental β-TCP compared to other bone substitutes. Acta Scientiarum. Health Sci..

[24] Zanatta M., Valenti M.T., Donatelli L., Zucal C., Carbonare L.D. (2012). Runx-2 gene expression is associated with age-related changes of bone mineral density in the healthy young-adult population. J. Bone Miner. Metab..

[25] Hu K., Olsen B.R. (2016). The roles of vascular endothelial growth factor in bone repair and regeneration. Bone.

[26] Schulz K.F., Altman D.G., Moher D., Group C. (2010). CONSORT 2010 Statement: Updated guidelines for reporting parallel group randomised trials. Trials.

[27] Pereira R.S., Pavelski M.D., Griza G.L., Boos F., Hochuli-Vieira E. (2019). Prospective evaluation of morbidity in patients who underwent autogenous bone-graft harvesting from the mandibular symphysis and retromolar regions. Clin. Implant Dent. Relat. Res..

[28] Cohen M.M. (2009). Perspectives on RUNX genes: An update. Am. J. Med. Genet. A.

[29] Street J., Bao M., DeGuzman L., Bunting S., Peale F.V., Ferrara N., Steinmetz H., Hoeffel J., Cleland J.L., Daugherty A. (2002). Vascular endothelial growth factor stimulates bone repair by promoting angiogenesis and bone turnover. Proc. Natl. Acad. Sci. USA.

[30] Pereira R.D., Menezes J.D., Bonardi J.P., Griza G.L., Okamoto R., Hochuli-Vieira E. (2017). Histomorphometric and immunohistochemical assessment of RUNX2 and VEGF of BiogranTM and autogenous bone graft in human maxillary sinus bone augmentation: A prospective and randomized study. Clin. Implant Dent. Relat. Res..

[31] Karageorgiou V., Kaplan D. (2005). Porosity of 3D biomaterial scaffolds and osteogenesis. Biomaterials.

[32] Yuan H., Kurashina K., de Bruijn J.D., Li Y., de Groot K., Zhang X. (1999). A preliminary study on osteoinduction of two kinds of calcium phosphate ceramics. Biomaterials.

[33] Khan S.N., Cammisa F.P., Sandhu H.S., Diwan A.D., Girardi F.P., Lane J.M. (2005). The biology of bone grafting. J. Am. Acad. Orthop. Surg..

[34] Avila G., Neiva R., Misch C.E., Galindo-Moreno P., Benavides E., Rudek I., Wang H.-L. (2010). Clinical and Histologic Outcomes After the Use of a Novel Allograft for Maxillary Sinus Augmentation: A Case Series. Implant Dent..

[35] Jang H.-Y., Kim H.-C., Lee S.-C., Lee J.-Y. (2010). Choice of Graft Material in Relation to Maxillary Sinus Width in Internal Sinus Floor Augmentation. J. Oral Maxillofac. Surg..

[36] Krennmair G., Krainhöfner M., Maier H., Weinländer M., Piehslinger E. (2006). Computerized tomography-assisted calculation of sinus augmentation volume. Int. J. Oral Maxillofac. Implant..

[37] Kempen D.H., Lu L., Heijink A., Hefferan T.E., Creemers L.B., Maran A., Yaszemski M.J., Dhert W.J. (2009). Effect of local sequential VEGF and BMP-2 delivery on ectopic and orthotopic bone regeneration. Biomaterials.

